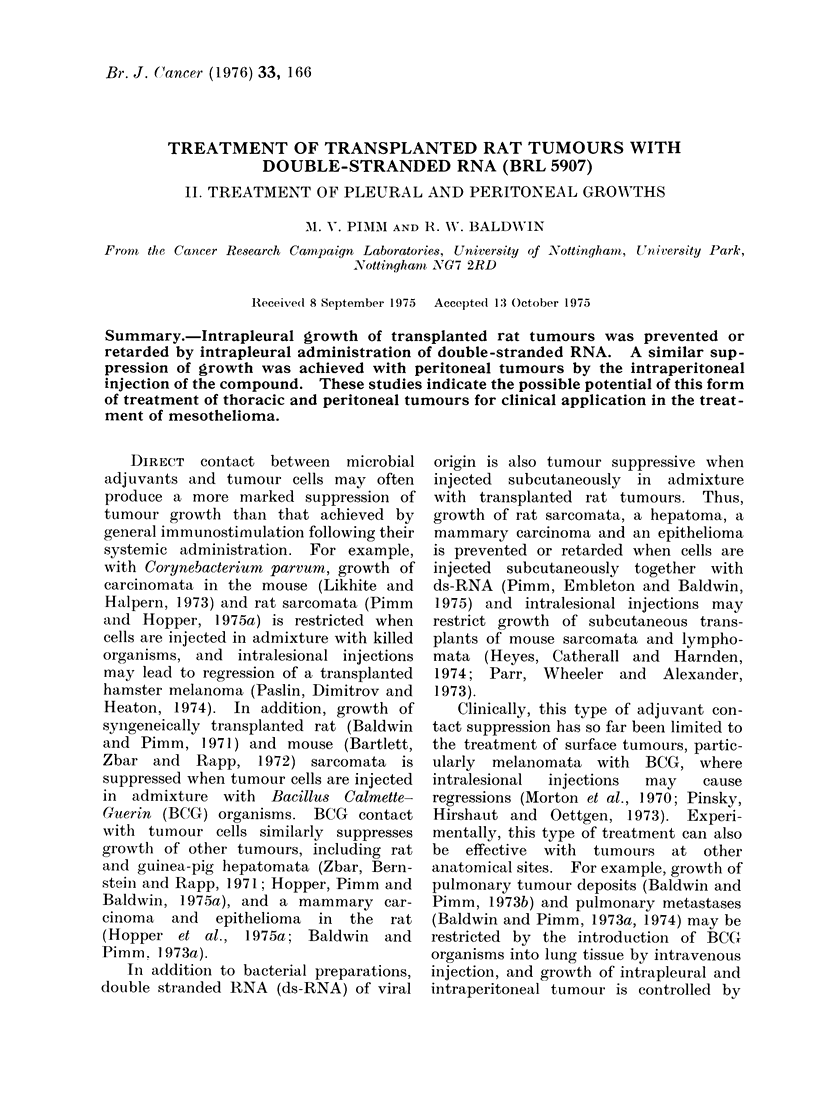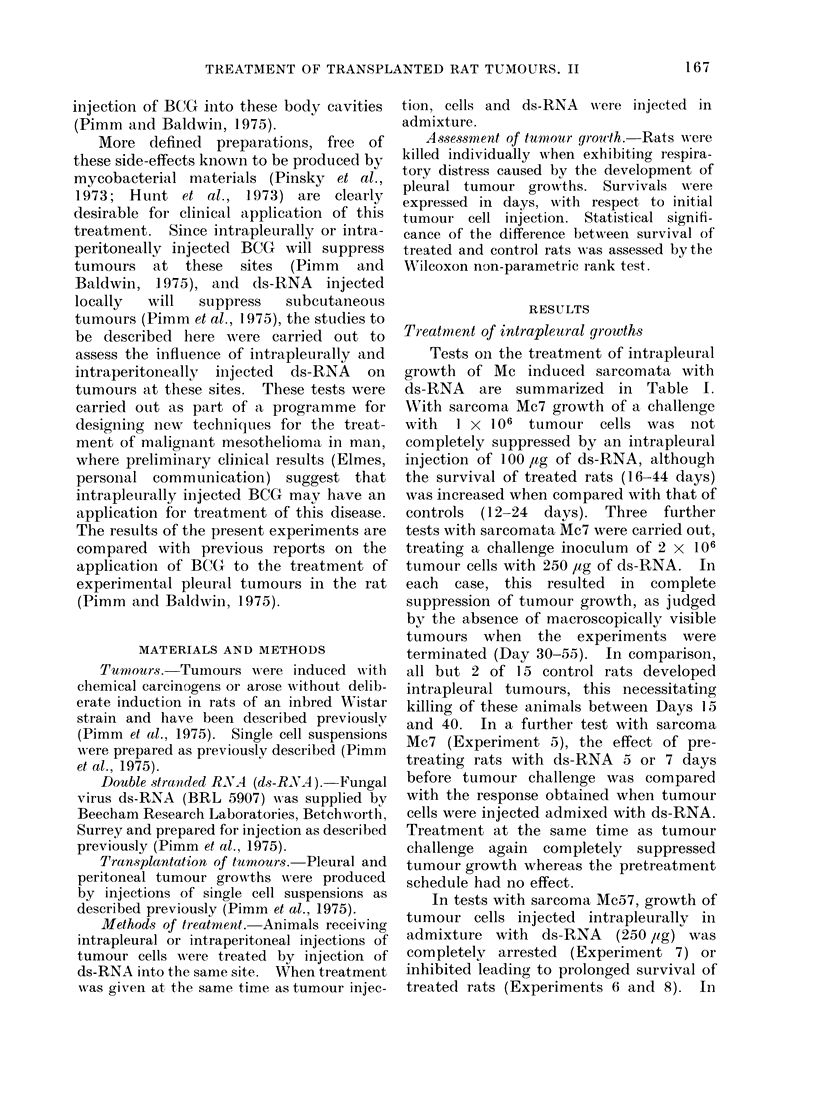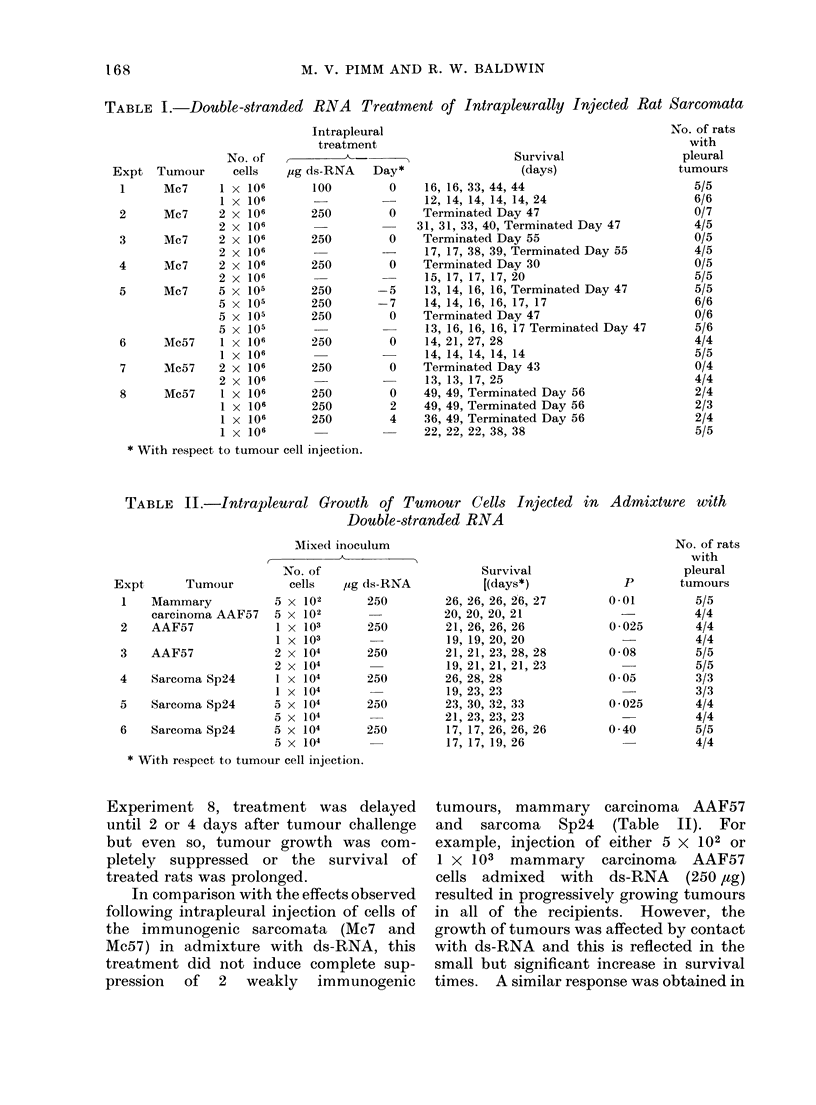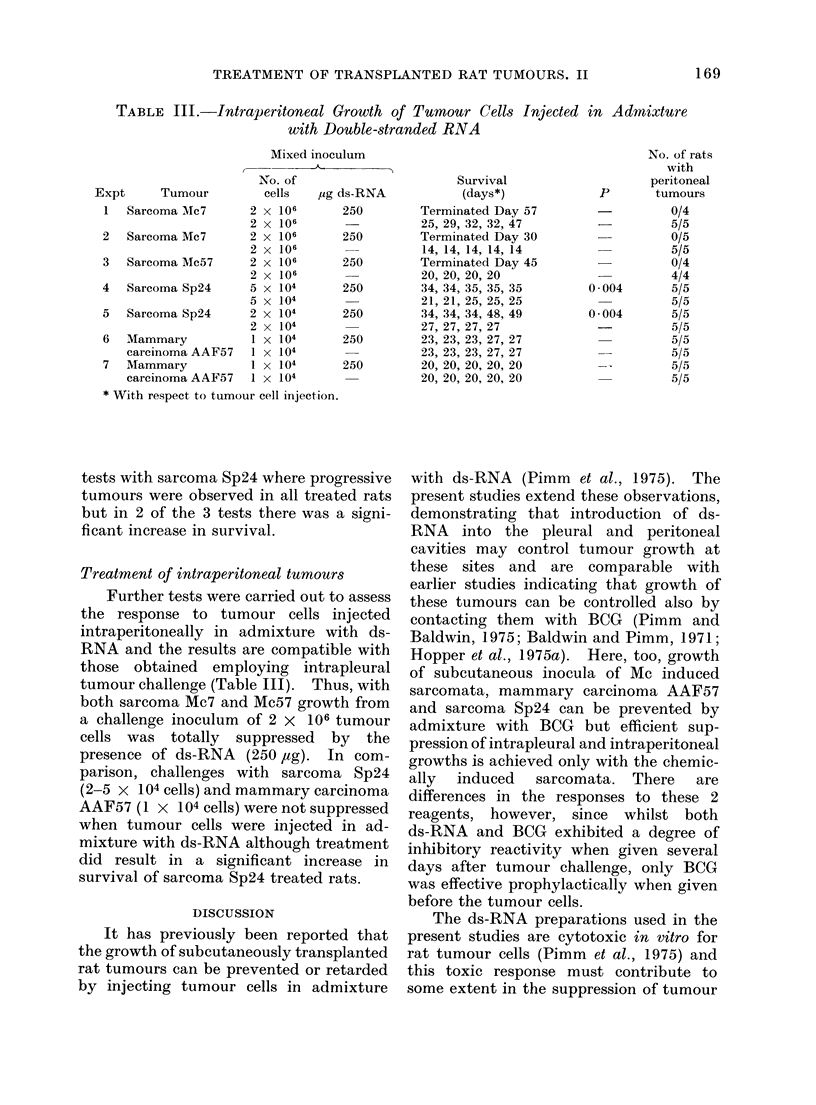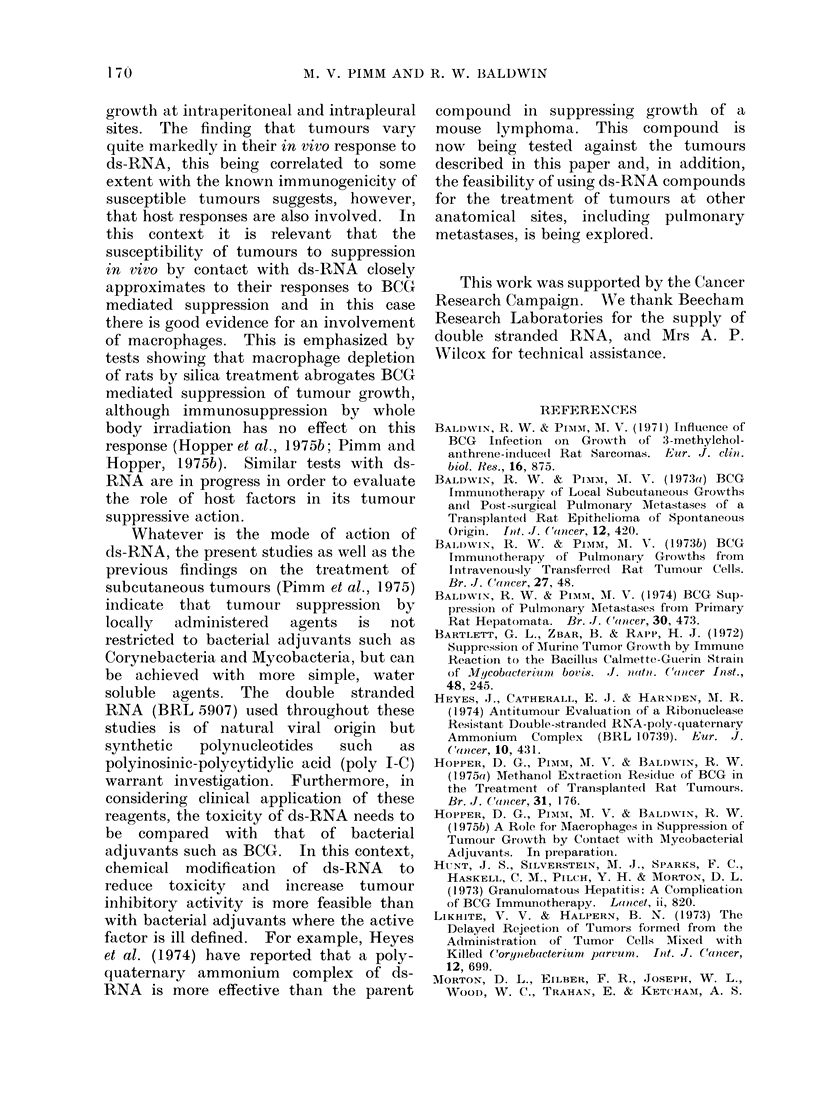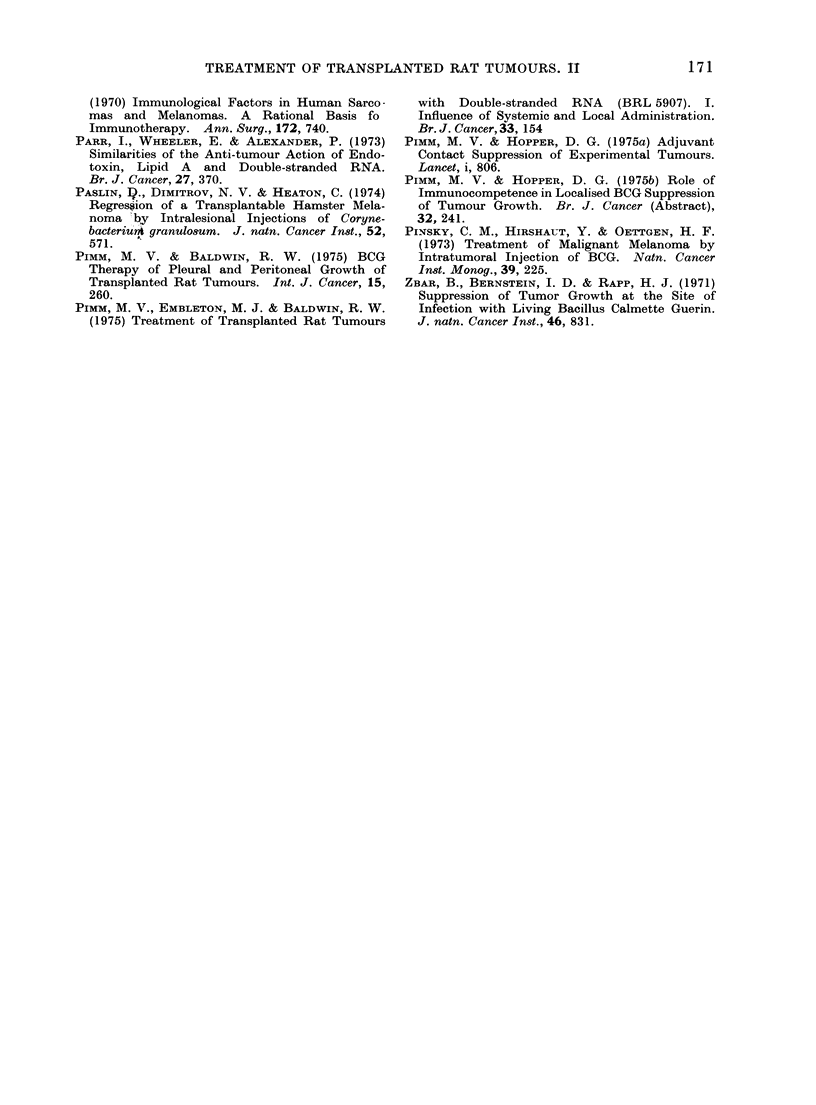# Treatment of transplanted rat tumours with double-stranded RNA(BRL 5907). II. Treatment of pleural and peritoneal growths.

**DOI:** 10.1038/bjc.1976.21

**Published:** 1976-02

**Authors:** M. V. Pimm, R. W. Baldwin

## Abstract

Intrapleural growth of transplanted rat tumours was prevented or retarded by intrapleural administration of double-stranded RNA. A similar suppression of growth was achieved with peitoneal tumours by the intraperitoneal injection of the compound. These studies indicate the possible potential of this form of treatment of thoracic and peritoneal tumours for clinical application in the treatment of mesothelioma.


Br. J. Cancer (1976) 33, 166

### TREATMENT OF TRANSPLANTED RAT TUMOURS WITH DOUBLE-STRANDED RNA (BRL 5907)

### II. TREATMENT OF PLEURAL AND PERITONEAL GROWTHS

M. V. PIMM AND R. W. BALDWIN

*From the Cancer Research Campaign Laboratories, University of Nottingham, University Park, Nottingham NG7 2RD*

Received 8 September 1975 Accepted 13 October 1975

**Summary.—Intrapleural growth of transplanted rat tumours was prevented or retarded by intrapleural administration of double-stranded RNA. A similar suppression of growth was achieved with peritoneal tumours by the intraperitoneal injection of the compound. These studies indicate the possible potential of this form of treatment of thoracic and peritoneal tumours for clinical application in the treatment of mesothelioma.**

DIRECT contact between microbial adjuvants and tumour cells may often produce a more marked suppression of tumour growth than that achieved by general immunostimulation following their systemic administration. For example, with *Corynebacterium parvum*, growth of carcinomata in the mouse (Likhite and Halpern, 1973) and rat sarcomata (Pimm and Hopper, 1975*a*) is restricted when cells are injected in admixture with killed organisms, and intralesional injections may lead to regression of a transplanted hamster melanoma (Paslin, Dimitrov and Heaton, 1974). In addition, growth of syngeneically transplanted rat (Baldwin and Pimm, 1971) and mouse (Bartlett, Zbar and Rapp, 1972) sarcomata is suppressed when tumour cells are injected in admixture with *Bacillus Calmette–Guerin* (BCG) organisms. BCG contact with tumour cells similarly suppresses growth of other tumours, including rat and guinea-pig hepatomata (Zbar, Bernstein and Rapp, 1971; Hopper, Pimm and Baldwin, 1975*a*), and a mammary carcinoma and epithelioma in the rat (Hopper *et al.*, 1975*a*; Baldwin and Pimm, 1973*a*).

In addition to bacterial preparations, double stranded RNA (ds-RNA) of viral origin is also tumour suppressive when injected subcutaneously in admixture with transplanted rat tumours. Thus, growth of rat sarcomata, a hepatoma, a mammary carcinoma and an epithelioma is prevented or retarded when cells are injected subcutaneously together with ds-RNA (Pimm, Embleton and Baldwin, 1975) and intralesional injections may restrict growth of subcutaneous transplants of mouse sarcomata and lymphomata (Heyes, Catherall and Harnden, 1974; Parr, Wheeler and Alexander, 1973).

Clinically, this type of adjuvant contact suppression has so far been limited to the treatment of surface tumours, particularly melanomata with BCG, where intralesional injections may cause regressions (Morton *et al.*, 1970; Pinsky, Hirshaut and Oettgen, 1973). Experimentally, this type of treatment can also be effective with tumours at other anatomical sites. For example, growth of pulmonary tumour deposits (Baldwin and Pimm, 1973*b*) and pulmonary metastases (Baldwin and Pimm, 1973*a*, 1974) may be restricted by the introduction of BCG organisms into lung tissue by intravenous injection, and growth of intrapleural and intraperitoneal tumour is controlled by injection of BCG into these body cavities (Pimm and Baldwin, 1975).

More defined preparations, free of these side-effects known to be produced by mycobacterial materials (Pinsky *et al.*, 1973; Hunt *et al.*, 1973) are clearly desirable for clinical application of this treatment. Since intrapleurally or intraperitoneally injected BCG will suppress tumours at these sites (Pimm and Baldwin, 1975), and ds-RNA injected locally will suppress subcutaneous tumours (Pimm *et al.*, 1975), the studies to be described here were carried out to assess the influence of intrapleurally and intraperitoneally injected ds-RNA on tumours at these sites. These tests were carried out as part of a programme for designing new techniques for the treatment of malignant mesothelioma in man, where preliminary clinical results (Elmes, personal communication) suggest that intrapleurally injected BCG may have an application for treatment of this disease. The results of the present experiments are compared with previous reports on the application of BCG to the treatment of experimental pleural tumours in the rat (Pimm and Baldwin, 1975).

### MATERIALS AND METHODS

*Tumours.*—Tumours were induced with chemical carcinogens or arose without deliberate induction in rats of an inbred Wistar strain and have been described previously (Pimm *et al.*, 1975). Single cell suspensions were prepared as previously described (Pimm *et al.*, 1975).

*Double stranded RNA (ds-RNA).*—Fungal virus ds-RNA (BRL 5907) was supplied by Beecham Research Laboratories, Betchworth, Surrey and prepared for injection as described previously (Pimm *et al.*, 1975).

*Transplantation of tumours.*—Pleural and peritoneal tumour growths were produced by injections of single cell suspensions as described previously (Pimm *et al.*, 1975).

*Methods of treatment.*—Animals receiving intrapleural or intraperitoneal injections of tumour cells were treated by injection of ds-RNA into the same site. When treatment was given at the same time as tumour injection, cells and ds-RNA were injected in admixture.

*Assessment of tumour growth.*—Rats were killed individually when exhibiting respiratory distress caused by the development of pleural tumour growths. Survivals were expressed in days, with respect to initial tumour cell injection. Statistical significance of the difference between survival of treated and control rats was assessed by the Wilcoxon non-parametric rank test.

### RESULTS

### *Treatment of intrapleural growths*

Tests on the treatment of intrapleural growth of Mc induced sarcomata with ds-RNA are summarized in Table I. With sarcoma Mc7 growth of a challenge with $1 \times 10^6$ tumour cells was not completely suppressed by an intrapleural injection of 100 $\mu$g of ds-RNA, although the survival of treated rats (16–44 days) was increased when compared with that of controls (12–24 days). Three further tests with sarcomata Mc7 were carried out, treating a challenge inoculum of $2 \times 10^6$ tumour cells with 250 $\mu$g of ds-RNA. In each case, this resulted in complete suppression of tumour growth, as judged by the absence of macroscopically visible tumours when the experiments were terminated (Day 30–55). In comparison, all but 2 of 15 control rats developed intrapleural tumours, this necessitating killing of these animals between Days 15 and 40. In a further test with sarcoma Mc7 (Experiment 5), the effect of pretreating rats with ds-RNA 5 or 7 days before tumour challenge was compared with the response obtained when tumour cells were injected admixed with ds-RNA. Treatment at the same time as tumour challenge again completely suppressed tumour growth whereas the pretreatment schedule had no effect.

In tests with sarcoma Mc57, growth of tumour cells injected intrapleurally in admixture with ds-RNA (250 $\mu$g) was completely arrested (Experiment 7) or inhibited leading to prolonged survival of treated rats (Experiments 6 and 8). In

TABLE I.—*Double-stranded RNA Treatment of Intrapleurally Injected Rat Sarcomata*

| Expt | Tumour | No. of cells | Intrapleural treatment: μg ds-RNA | Intrapleural treatment: Day* | Survival (days) | No. of rats with pleural tumours |
|---|---|---|---|---|---|---|
| 1 | Mc7 | $1 \times 10^6$ | 100 | 0 | 16, 16, 33, 44, 44 | 5/5 |
| | | $1 \times 10^6$ | — | — | 12, 14, 14, 14, 14, 24 | 6/6 |
| 2 | Mc7 | $2 \times 10^6$ | 250 | 0 | Terminated Day 47 | 0/7 |
| | | $2 \times 10^6$ | — | — | 31, 31, 33, 40, Terminated Day 47 | 4/5 |
| 3 | Mc7 | $2 \times 10^6$ | 250 | 0 | Terminated Day 55 | 0/5 |
| | | $2 \times 10^6$ | — | — | 17, 17, 38, 39, Terminated Day 55 | 4/5 |
| 4 | Mc7 | $2 \times 10^6$ | 250 | 0 | Terminated Day 30 | 0/5 |
| | | $2 \times 10^6$ | — | — | 15, 17, 17, 17, 20 | 5/5 |
| 5 | Mc7 | $5 \times 10^5$ | 250 | −5 | 13, 14, 16, 16, Terminated Day 47 | 5/5 |
| | | $5 \times 10^5$ | 250 | −7 | 14, 14, 16, 16, 17, 17 | 6/6 |
| | | $5 \times 10^5$ | 250 | 0 | Terminated Day 47 | 0/6 |
| | | $5 \times 10^5$ | — | — | 13, 16, 16, 16, 17 Terminated Day 47 | 5/6 |
| 6 | Mc57 | $1 \times 10^6$ | 250 | 0 | 14, 21, 27, 28 | 4/4 |
| | | $1 \times 10^6$ | — | — | 14, 14, 14, 14, 14 | 5/5 |
| 7 | Mc57 | $2 \times 10^6$ | 250 | 0 | Terminated Day 43 | 0/4 |
| | | $2 \times 10^6$ | — | — | 13, 13, 17, 25 | 4/4 |
| 8 | Mc57 | $1 \times 10^6$ | 250 | 0 | 49, 49, Terminated Day 56 | 2/4 |
| | | $1 \times 10^6$ | 250 | 2 | 49, 49, Terminated Day 56 | 2/3 |
| | | $1 \times 10^6$ | 250 | 4 | 36, 49, Terminated Day 56 | 2/4 |
| | | $1 \times 10^6$ | — | — | 22, 22, 22, 38, 38 | 5/5 |

* With respect to tumour cell injection.

TABLE II.—*Intrapleural Growth of Tumour Cells Injected in Admixture with Double-stranded RNA*

| Expt | Tumour | Mixed inoculum: No. of cells | Mixed inoculum: μg ds-RNA | Survival (days*) | P | No. of rats with pleural tumours |
|---|---|---|---|---|---|---|
| 1 | Mammary | $5 \times 10^2$ | 250 | 26, 26, 26, 26, 27 | 0·01 | 5/5 |
| | carcinoma AAF57 | $5 \times 10^2$ | — | 20, 20, 20, 21 | — | 4/4 |
| 2 | AAF57 | $1 \times 10^3$ | 250 | 21, 26, 26, 26 | 0·025 | 4/4 |
| | | $1 \times 10^3$ | — | 19, 19, 20, 20 | — | 4/4 |
| 3 | AAF57 | $2 \times 10^4$ | 250 | 21, 21, 23, 28, 28 | 0·08 | 5/5 |
| | | $2 \times 10^4$ | — | 19, 21, 21, 21, 23 | — | 5/5 |
| 4 | Sarcoma Sp24 | $1 \times 10^4$ | 250 | 26, 28, 28 | 0·05 | 3/3 |
| | | $1 \times 10^4$ | — | 19, 23, 23 | — | 3/3 |
| 5 | Sarcoma Sp24 | $5 \times 10^4$ | 250 | 23, 30, 32, 33 | 0·025 | 4/4 |
| | | $5 \times 10^4$ | — | 21, 23, 23, 23 | — | 4/4 |
| 6 | Sarcoma Sp24 | $5 \times 10^4$ | 250 | 17, 17, 26, 26, 26 | 0·40 | 5/5 |
| | | $5 \times 10^4$ | — | 17, 17, 19, 26 | — | 4/4 |

* With respect to tumour cell injection.

Experiment 8, treatment was delayed until 2 or 4 days after tumour challenge but even so, tumour growth was completely suppressed or the survival of treated rats was prolonged.

In comparison with the effects observed following intrapleural injection of cells of the immunogenic sarcomata (Mc7 and Mc57) in admixture with ds-RNA, this treatment did not induce complete suppression of 2 weakly immunogenic tumours, mammary carcinoma AAF57 and sarcoma Sp24 (Table II). For example, injection of either $5 \times 10^2$ or $1 \times 10^3$ mammary carcinoma AAF57 cells admixed with ds-RNA (250 μg) resulted in progressively growing tumours in all of the recipients. However, the growth of tumours was affected by contact with ds-RNA and this is reflected in the small but significant increase in survival times. A similar response was obtained in

TABLE III.—*Intraperitoneal Growth of Tumour Cells Injected in Admixture with Double-stranded RNA*

| Expt | Tumour | Mixed inoculum: No. of cells | Mixed inoculum: μg ds-RNA | Survival (days*) | P | No. of rats with peritoneal tumours |
|---|---|---|---|---|---|---|
| 1 | Sarcoma Mc7 | $2 \times 10^6$ | 250 | Terminated Day 57 | — | 0/4 |
| | | $2 \times 10^6$ | — | 25, 29, 32, 32, 47 | — | 5/5 |
| 2 | Sarcoma Mc7 | $2 \times 10^6$ | 250 | Terminated Day 30 | — | 0/5 |
| | | $2 \times 10^6$ | — | 14, 14, 14, 14, 14 | — | 5/5 |
| 3 | Sarcoma Mc57 | $2 \times 10^6$ | 250 | Terminated Day 45 | — | 0/4 |
| | | $2 \times 10^6$ | — | 20, 20, 20, 20 | — | 4/4 |
| 4 | Sarcoma Sp24 | $5 \times 10^4$ | 250 | 34, 34, 35, 35, 35 | 0·004 | 5/5 |
| | | $5 \times 10^4$ | — | 21, 21, 25, 25, 25 | — | 5/5 |
| 5 | Sarcoma Sp24 | $2 \times 10^4$ | 250 | 34, 34, 34, 48, 49 | 0·004 | 5/5 |
| | | $2 \times 10^4$ | — | 27, 27, 27, 27 | — | 5/5 |
| 6 | Mammary carcinoma AAF57 | $1 \times 10^4$ | 250 | 23, 23, 23, 27, 27 | — | 5/5 |
| | | $1 \times 10^4$ | — | 23, 23, 23, 27, 27 | — | 5/5 |
| 7 | Mammary carcinoma AAF57 | $1 \times 10^4$ | 250 | 20, 20, 20, 20, 20 | — | 5/5 |
| | | $1 \times 10^4$ | — | 20, 20, 20, 20, 20 | — | 5/5 |

* With respect to tumour cell injection.

tests with sarcoma Sp24 where progressive tumours were observed in all treated rats but in 2 of the 3 tests there was a significant increase in survival.

*Treatment of intraperitoneal tumours*

Further tests were carried out to assess the response to tumour cells injected intraperitoneally in admixture with ds-RNA and the results are compatible with those obtained employing intrapleural tumour challenge (Table III). Thus, with both sarcoma Mc7 and Mc57 growth from a challenge inoculum of $2 \times 10^6$ tumour cells was totally suppressed by the presence of ds-RNA (250 μg). In comparison, challenges with sarcoma Sp24 (2–5 $\times 10^4$ cells) and mammary carcinoma AAF57 ($1 \times 10^4$ cells) were not suppressed when tumour cells were injected in admixture with ds-RNA although treatment did result in a significant increase in survival of sarcoma Sp24 treated rats.

### DISCUSSION

It has previously been reported that the growth of subcutaneously transplanted rat tumours can be prevented or retarded by injecting tumour cells in admixture with ds-RNA (Pimm *et al.*, 1975). The present studies extend these observations, demonstrating that introduction of ds-RNA into the pleural and peritoneal cavities may control tumour growth at these sites and are comparable with earlier studies indicating that growth of these tumours can be controlled also by contacting them with BCG (Pimm and Baldwin, 1975; Baldwin and Pimm, 1971; Hopper *et al.*, 1975*a*). Here, too, growth of subcutaneous inocula of Mc induced sarcomata, mammary carcinoma AAF57 and sarcoma Sp24 can be prevented by admixture with BCG but efficient suppression of intrapleural and intraperitoneal growths is achieved only with the chemically induced sarcomata. There are differences in the responses to these 2 reagents, however, since whilst both ds-RNA and BCG exhibited a degree of inhibitory reactivity when given several days after tumour challenge, only BCG was effective prophylactically when given before the tumour cells.

The ds-RNA preparations used in the present studies are cytotoxic *in vitro* for rat tumour cells (Pimm *et al.*, 1975) and this toxic response must contribute to some extent in the suppression of tumour growth at intraperitoneal and intrapleural sites. The finding that tumours vary quite markedly in their *in vivo* response to ds-RNA, this being correlated to some extent with the known immunogenicity of susceptible tumours suggests, however, that host responses are also involved. In this context it is relevant that the susceptibility of tumours to suppression *in vivo* by contact with ds-RNA closely approximates to their responses to BCG mediated suppression and in this case there is good evidence for an involvement of macrophages. This is emphasized by tests showing that macrophage depletion of rats by silica treatment abrogates BCG mediated suppression of tumour growth, although immunosuppression by whole body irradiation has no effect on this response (Hopper *et al.*, 1975*b*; Pimm and Hopper, 1975*b*). Similar tests with ds-RNA are in progress in order to evaluate the role of host factors in its tumour suppressive action.

Whatever is the mode of action of ds-RNA, the present studies as well as the previous findings on the treatment of subcutaneous tumours (Pimm *et al.*, 1975) indicate that tumour suppression by locally administered agents is not restricted to bacterial adjuvants such as Corynebacteria and Mycobacteria, but can be achieved with more simple, water soluble agents. The double stranded RNA (BRL 5907) used throughout these studies is of natural viral origin but synthetic polynucleotides such as polyinosinic-polycytidylic acid (poly I-C) warrant investigation. Furthermore, in considering clinical application of these reagents, the toxicity of ds-RNA needs to be compared with that of bacterial adjuvants such as BCG. In this context, chemical modification of ds-RNA to reduce toxicity and increase tumour inhibitory activity is more feasible than with bacterial adjuvants where the active factor is ill defined. For example, Heyes *et al.* (1974) have reported that a poly-quaternary ammonium complex of ds-RNA is more effective than the parent compound in suppressing growth of a mouse lymphoma. This compound is now being tested against the tumours described in this paper and, in addition, the feasibility of using ds-RNA compounds for the treatment of tumours at other anatomical sites, including pulmonary metastases, is being explored.

This work was supported by the Cancer Research Campaign. We thank Beecham Research Laboratories for the supply of double stranded RNA, and Mrs A. P. Wilcox for technical assistance.

REFERENCES

Baldwin, R. W. & Pimm, M. V. (1971) Influence of BCG Infection on Growth of 3-methylcholanthrene-induced Rat Sarcomas. *Eur. J. clin. biol. Res.*, **16**, 875.

Baldwin, R. W. & Pimm, M. V. (1973*a*) BCG Immunotherapy of Local Subcutaneous Growths and Post-surgical Pulmonary Metastases of a Transplanted Rat Epithelioma of Spontaneous Origin. *Int. J. Cancer*, **12**, 420.

Baldwin, R. W. & Pimm, M. V. (1973*b*) BCG Immunotherapy of Pulmonary Growths from Intravenously Transferred Rat Tumour Cells. *Br. J. Cancer*, **27**, 48.

Baldwin, R. W. & Pimm, M. V. (1974) BCG Suppression of Pulmonary Metastases from Primary Rat Hepatomata. *Br. J. Cancer*, **30**, 473.

Bartlett, G. L., Zbar, B. & Rapp, H. J. (1972) Suppression of Murine Tumor Growth by Immune Reaction to the Bacillus Calmette-Guerin Strain of *Mycobacterium bovis*. *J. natn. Cancer Inst.*, **48**, 245.

Heyes, J., Catherall, E. J. & Harnden, M. R. (1974) Antitumour Evaluation of a Ribonuclease Resistant Double-stranded RNA-poly-quaternary Ammonium Complex (BRL 10739). *Eur. J. Cancer*, **10**, 431.

Hopper, D. G., Pimm, M. V. & Baldwin, R. W. (1975*a*) Methanol Extraction Residue of BCG in the Treatment of Transplanted Rat Tumours. *Br. J. Cancer*, **31**, 176.

Hopper, D. G., Pimm, M. V. & Baldwin, R. W. (1975*b*) A Role for Macrophages in Suppression of Tumour Growth by Contact with Mycobacterial Adjuvants. In preparation.

Hunt, J. S., Silverstein, M. J., Sparks, F. C., Haskell, C. M., Pilch, Y. H. & Morton, D. L. (1973) Granulomatous Hepatitis: A Complication of BCG Immunotherapy. *Lancet*, ii, 820.

Likhite, V. V. & Halpern, B. N. (1973) The Delayed Rejection of Tumors formed from the Administration of Tumor Cells Mixed with Killed *Corynebacterium parvum*. *Int. J. Cancer*, **12**, 699.

Morton, D. L., Eilber, F. R., Joseph, W. L., Wood, W. C., Trahan, E. & Ketcham, A. S.

(1970) Immunological Factors in Human Sarcomas and Melanomas. A Rational Basis fo Immunotherapy. *Ann. Surg.*, **172**, 740.

PARR, I., WHEELER, E. & ALEXANDER, P. (1973) Similarities of the Anti-tumour Action of Endotoxin, Lipid A and Double-stranded RNA. *Br. J. Cancer*, **27**, 370.

PASLIN, D., DIMITROV, N. V. & HEATON, C. (1974) Regression of a Transplantable Hamster Melanoma by Intralesional Injections of *Corynebacterium granulosum*. *J. natn. Cancer Inst.*, **52**, 571.

PIMM, M. V. & BALDWIN, R. W. (1975) BCG Therapy of Pleural and Peritoneal Growth of Transplanted Rat Tumours. *Int. J. Cancer*, **15**, 260.

PIMM, M. V., EMBLETON, M. J. & BALDWIN, R. W. (1975) Treatment of Transplanted Rat Tumours with Double-stranded RNA (BRL 5907). I. Influence of Systemic and Local Administration. *Br. J. Cancer*, **33**, 154

PIMM, M. V. & HOPPER, D. G. (1975*a*) Adjuvant Contact Suppression of Experimental Tumours. *Lancet*, i, 806.

PIMM, M. V. & HOPPER, D. G. (1975*b*) Role of Immunocompetence in Localised BCG Suppression of Tumour Growth. *Br. J. Cancer* (Abstract), **32**, 241.

PINSKY, C. M., HIRSHAUT, Y. & OETTGEN, H. F. (1973) Treatment of Malignant Melanoma by Intratumoral Injection of BCG. *Natn. Cancer Inst. Monog.*, **39**, 225.

ZBAR, B., BERNSTEIN, I. D. & RAPP, H. J. (1971) Suppression of Tumor Growth at the Site of Infection with Living Bacillus Calmette Guerin. *J. natn. Cancer Inst.*, **46**, 831.